# Research on crack propagation behaviour of EB-PVD TBCs based on TGO evolution

**DOI:** 10.1038/s41598-023-44878-x

**Published:** 2023-10-16

**Authors:** Lulu Wang, Jinying Zhan, Yankuan Liu, Yuansheng Wang, Akinola Ajayi, Zhiping Wang

**Affiliations:** 1 Shenyang University of Technology, Shenyang, 110870 China; 2 China Southern Airlines, Shenyang, 110169 China; 3 Tianjin Key Laboratory of Civil Aircraft Airworthiness and Maintenance, Civil Aviation University of China, Tianjin, 300300 China

**Keywords:** Engineering, Materials science

## Abstract

Thermal Barrier Coatings (TBCs) are functional coatings used to protect high-temperature components that are prone to early damage and premature failure under the influence of complex working conditions. This paper examines the crack propagation behaviour of 8% yttria-stabilized zirconia (8YSZ) EB-PVD TBCs under different oxidation conditions at 1100 °C. The morphology of interfacial cracks after oxidation was summarized and the evolution of thermally grown oxide (TGO) was quantified. Based on the evolution of TGO, the causes of crack propagation were analyzed. For the specimens after oxidation experiment, the interfacial crack propagation behaviour was observed and analyzed by SEM, and the reason of lateral crack propagation was explained from the perspective of interfacial fracture toughness. The reason for crack deflection is analyzed from the perspective of energy release rate. The equivalent thickness, normalized rumpling index and two-dimensional roughness index were calculated, then the TGO growth behaviour was comprehensively analyzed and related to the crack propagation.

## Introduction

Thermal barrier coatings (TBCs) are functional coatings used to protect high-temperature components and extend their service life, which have been used for many years on the surfaces of advanced gas turbines, aero-engine blades and other related components^1,2^. TBCs layers generally consist of a ceramic top coat (TC) and metal bond coat (BC). Currently, the most widely used material is MCrAlY (M=Ni, Co, or Ni + Co) as the bond coat, and 6 ~ 8wt% Y_2_O_3_ stabilized ZrO_2_ (yttrium-stabilized zirconia, YSZ) as the top coat, which has a high melting point (about 2700 °C) and low thermal conductivity (the dense material is about 2.3 W·(m K)^−1^) at 1000 °C)^3–6^. Thermally grown oxides (TGO) form between the TC and BC due to thermal exposure. TGOs' structure and composition change as high-temperature oxidation progress, which seriously affects the mechanical properties and service life of TBCs^7^.

In a high-temperature environment, oxygen reacts with Al, Co, and Ni in the metal bond coat, causing a change at the interface and internal oxidation. The increase in the oxidation degree leads to the formation and evolution of Al_2_O_3,_ layering of TGO (with α- Al2O_3_ and Cr_2_O_3_), formation of spinel phases (Ni(Al, Cr)_2_O_4_ and NiO), and precipitation of elemental metallic phases (γ-Ni, α-Cr, γ-Al, β-Ti)^8^.

Under the influence of thermal oxidation and other mechanical behaviors, TGO will be subjected to in-plane compressive stress, resulting in out-of-plane displacement, wrinkling and forming defects. At the same time, the adhesive layer can be deformed by ratcheting and mechanical stresses such as subsidence and cavity formation. These deformations create tensile stress perpendicular to the interface and cause delamination of the TBCs^9^. The stress perpendicular to the interface in the defect will cause cracks to form. The further development of these cracks will lead to a greater degree of stratification in the weak area of the interface.

Thus it can be seen that the TGO is one of the major failure factors of TBCs and has attracted much attention from researchers^10^. At present, some research results have been achieved by using the external load for experimental methods, layered structures for analyzing TGO kinetics, numerical analysis and other theories for calculation, and finite element software for simulation, etc. Doleker and Ozgurluk et al.^7,11,12^ have conducted a series of researches on studying the TGO growth and kinetic behaviors of different ceramic top coat structures, results show that coatings composed of lanthanide oxide such as La_2_Zr_2_O_7_ and Gd2Zr_2_O_7_ exhibit better performance because of the lower oxygen permeability. While Bal et al.^13,14^ proved that CMAS (CaO_2_, MgO_2_, Al_2_O_3_, SiO_2_) and hot corrosion to the TBCs can also bring a negative influence on the TGO kinetic behaviour and interfacial bonding strength. TGO growing process compared by different thermal oxidation temperatures has also been analyzed by Parlakyigit et al.^15^. It has been revealed that for TBCs with a NiCr bond coat, TGO grows more rapidly at 1100 °C than at 900–1000 °C, at the same time presenting more complex elemental composition in the TGO layer.

However, as the thickness of TGO is very small and the shape is irregular, the relationship between TGO growth kinetics and crack propagation behavior needs to be further studied. Thus, based on the thermal oxidation experiment, this paper aims to analyze the law of TBCs interfacial crack derivation according to the actual morphology of the internal cross-section and explore the influence of TGO evolution on the trend of interfacial crack derivation and propagation during thermal oxidation treatment.

## Experimental

### Material of specimens

**Figure 1:**
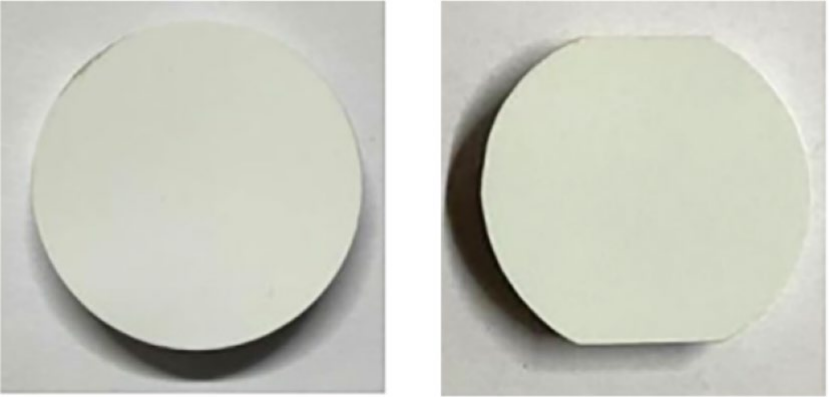
(**a**) Complete cylinder (**b**) Cylinder containing chord.

The substrates were cylindrical with a diameter of 25.4 mm and a thickness of 6 mm. Some specimens were prepared using two parallel chords with a chord length of 10 mm, as shown in Fig. 1a and b, respectively. The substrate material used was a nickel-based alloy-718. CoCrAlY was used to prepare the bond coat with a thickness of about 50 μm. The CoCrAlY powder was composed of 23 wt% of chromium (Cr), 13 wt% of aluminum (Al), 0.5 wt% of yttrium (Y) and balance cobalt (Co). YSZ was used to deposit the top coat, the thickness of this layer was approximately 100 μm. The typical equilibrium YSZ component is 92 wt% of ZrO_2_ and 8 wt% of Y_2_O_3_.

### Preparation of specimens

Specimens were prepared by EB-PVD. Before deposition, the surface of the substrate was sandblasted to ensure a better bonding strength between the substrate and the bond coat. The substrate was then pre-heated in a vacuum environment at 900 °C for 45 min, where the vacuum chamber pressure was 0.027–0.4 Pa, and then the CoCrAlY and YSZ were deposited sequentially.

### Oxidation tests condition

Isothermal oxidation experiment, cyclic oxidation experiment and mixed oxidation experiment were carried out at 1100 °C. For the isothermal oxidation experimental group, the specimens were treated for 25 h, 50 h, 75 h and 100 h respectively, and then cooled to room temperature in air. For the cyclic oxidation experimental group, the heat treatment for 1 h + air cooling for 15 min was used as a group of cycles, and the cycles were 25, 50, 75 times, respectively. For the mixed experimental group, the specimens in the group were first isothermally oxidized for 10 h to generate TGO, and then 5, 10, 15, 20, 25, 30, 35, and 40 cycles were performed respectively.

### Property characterizations

In order to better observe the cross-sectional microstructure before and after isothermal heat treatment and obtain accurate mechanical properties data, the specimens were cold-mounted by using epoxy resin and then coarse polished by 240, 400, 600, 800, 1000 and 1200 mesh silicon carbide papers, finally fine polishing was conducted by using 3.25、1.5 μm diamond polishing liquid, respectively.

The metallographic surface was sprayed with gold first to increase the conductivity, which can effectively improve the clarity and quality of SEM images. Then, Scanning Electron Microscopy (SEM, SIGMA 300, ZEISS, Germany) was used to observe the cross-sectional microstructure, crack propagation, and the TGO morphology. At the same time, the morphology of TGO was measured by software Image J. Energy dispersive spectroscopy (EDS, Oxford struments, Oxford, UK) was used to obtain the spatial distribution information of chemical element composition.

**Figure 2:**
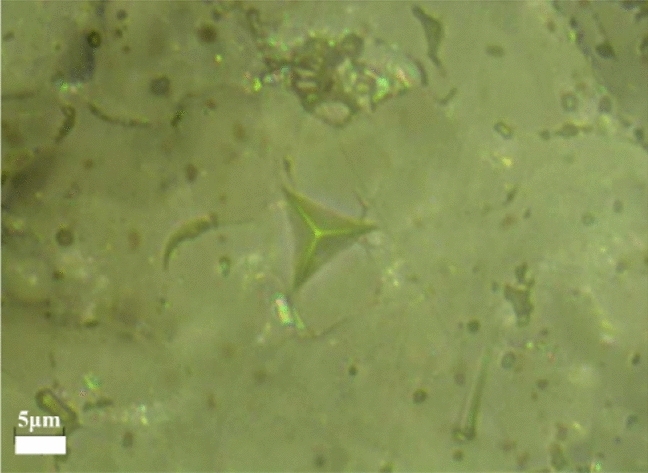
Imprint of the nano-indentation (Berkovich).

The Young’s modulus of TC, BC, TGO were measured by the nano-indentation method respectively. Like Fig. 2, indentations were produced by the CSM Indentation Tester (NHTX, CSM, Switzerland) equipped with a nano-indenter (Berkovich indenter). The measurement principle is based on the depth of the material to control the indentation. Hardness and Young’s modulus were calculated automatically by measuring the indentation size and the curve slope after the stress release. In addition, to ensure the accuracy of measuring results, Vickers Microhardness Tester and CSM Indentation Tester are used for 5–7 times of measurements on different kinds of specimens.

### Calculation of interfacial fracture toughness

Fracture toughness refers to the impedance value of the material that can resist fracture. It can be expressed by a single parameter describing the mechanical state of the crack tip, such as energy release rate G, stress intensity factor K, crack tip opening displacement or J-integral^16^. The value is only related to the material itself and the preparation process. When the crack size is constant, the greater the fracture toughness, the greater the critical stress required for crack instability propagation. When the stress is constant, the larger the fracture toughness, the larger the size derived when the crack reaches instability propagation.

**Figure 3:**
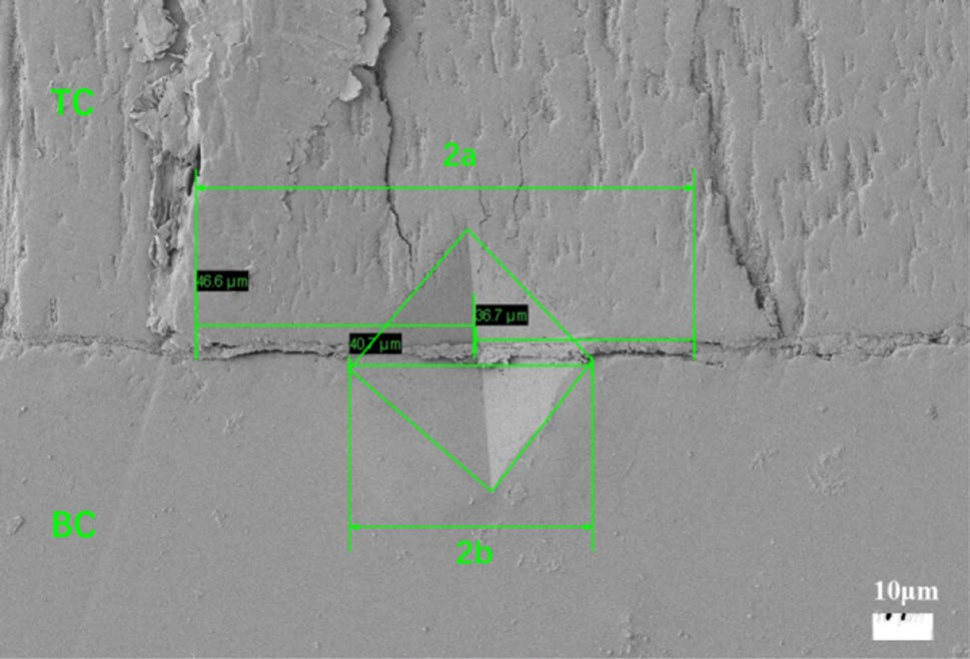
Typical schematic diagram of precise indentation at the TC/BC interface and the principle for measuring 2a (crack size) and 2b (indentation imprint size).

The stress intensity factor K is controlled by the stress perpendicular to the substrate and around the defect, which is an important index to judge whether the crack enters the instability. If the factor K of the cracked elastomer reaches or exceeds the critical fracture toughness K_c_ of the material when the unstable growth occurs at the crack tip, the unstable growth of the crack occurs and leads to the fracture of the cracked elastomer^17^. By calculating the fracture toughness of materials, the ability of materials to resist crack derivation can be well evaluated, and the direction of crack propagation can also be predicted. Based on previous studies, fracture toughness of interface was measured by indentation testing. This method is suitable for TBCs^18–22^. Figure 3 is a typical schematic diagram of precise indentation at the TC/BC and the crack propagate along with the interface, where *2b* is the diagonal length of the indentation and *a*_*c*_ is the distance from the crack tip to the indentation center. For a specimen corresponding to a given isothermal heat treatment condition, each interfacial indentation load *P* produces a square indentation with diagonal *2b*, and if the applied load P is equal or greater than the critical load *P*_*c*_ (*P* ≥ *P*_*c*_), a crack of size *a* (*a* ≥ *a*_*c*_) will be generated. In the experiment, the distance between the two crack tips on the diagonal *2a*_*c*_ is usually measured. In this experiment, five different loads of 0.98N, 1.96N, 2.94N, 4.9N and 9.8N were set up, and 4–6 indentations were made under each load to provide more calculation data.

It should be noted that *P*_*c*_ and corresponding critical crack length *a*_*c*_ are not determined by direct measurement, but in the form of fitting functions. That is, the intersection coordinates of apparent hardness line ln(b)-ln(P) (evolution of indentation size relative to the indentation force (master curve)) and ln(a) − ln(P) (the evolution of the crack size relative to the load force).

The hardness (Hv) of the specimens can be calculated according to the Eq. (1):

1 $$Hv=1.8544\frac{p}{{d}^{2}}$$

where the diagonal length *d* = *2b*, after its transformation can obtain:

2 $$ln\left(d\right)=0.5\mathit{ln}\left(P\right)+0.5\mathit{ln}\left(1.8544/{H}_{v}\right)$$

For bi-materials, Chicot et al.^23^ established a model, that the interface is locally treated as a homogeneous material, whose plastic and elastic properties would be generated by the respective contributions of top coat and bond coat. Therefore, the apparent toughness (*K*_*ca*_) of interface can be calculated by using following equation:

3 $${K}_{ca}=0.015{\left(\frac{E}{H}\right)}_{I}^{1/2}\frac{{P}_{c}}{{a}_{c}^{3/2}}$$

where *H* is the hardness, *E* is the Young's modulus (subscript *I* represents the interface), *P*_*c*_ is the critical value (N) of the load *P* applied by Vickers indentation at the interface between two materials of the system, *a*_*c*_ is the critical crack size (m). *P*_*c*_ and *a*_*c*_ correspond to the critical values of load and crack size, respectively, associated with the initiation of an interfacial crack caused by indentation.

In order to combine TC and BC into a bi-material system, ${\left(\frac{E}{H}\right)}_{I}^{1/2}$ in Eq. (3) is replaced by Eq. (4):

4 $$\left( \frac{E}{H} \right)_{I}^{1/2} = \frac{{\left( \frac{E}{H} \right)_{TC}^{1/2} }}{{1 + \left( {\frac{{H_{TC} }}{{H_{BC} }}} \right)^{1/2} }} + \frac{{\left( \frac{E}{H} \right)_{BC}^{1/2} }}{{1 + \left( {\frac{{H_{BC} }}{{H_{TC} }}} \right)^{1/2} }}$$

Considering that the TGO layer will affect the stability of TBCs, the fracture toughness of TGO/BC and TC/BC interfaces will be calculated and compared.

### Calculation of energy release rate

The energy release rate G is the energy released per unit thickness as the crack propagates per unit length in the plane. It mainly represents the toughness of the control failure plane of the coating and substrate. Furthermore, G is a function of phase angle, and the range of cracks that can be evaluated is wider.

**Figure 4:**
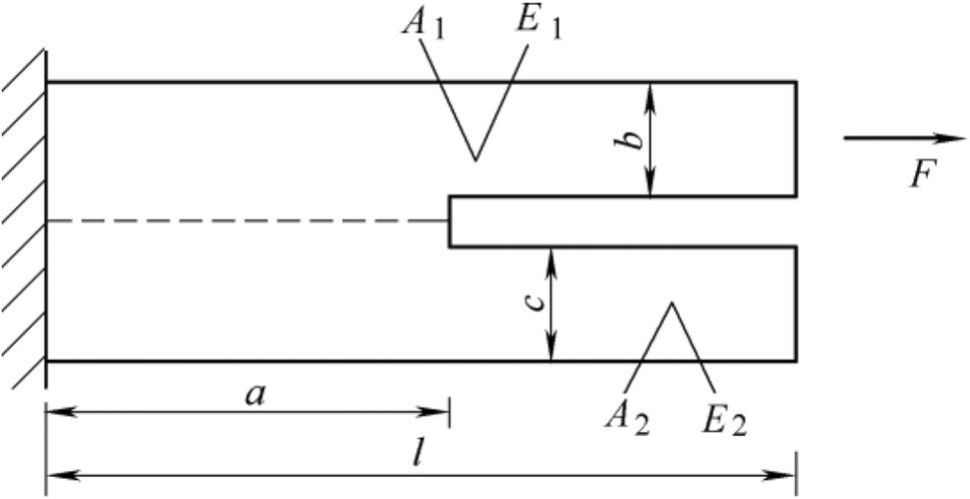
Schematic of the external force model of the bi-material system.

Before evaluating the crack, the energy of the system must be discussed first, according to the research of Li et al.^24^, the interface crack propagation model of bi-material under the influence of external force is shown in Fig. 4.

The total potential energy required for this system is:

5 $${E}_{p}=-\frac{F}{2}\frac{(l-a){A}_{2}{F}_{2}+l{A}_{1}{E}_{1}}{{A}_{1}{E}_{1}({A}_{1}{E}_{1}+{A}_{1}{E}_{2})}$$

where *E*_*p*_ represents the total potential energy required by the system,* F* is the magnitude of the external force that triggers crack propagation, *a* is the length of the uncracked area of the material, and *l* is the total length designed for the bi-material crack propagation model, *A* represents the cross-sectional area of the coating within the crack propagation range, *E* represents the Young’s modulus of the material, and subscripts 1 and 2 represent different materials in the bi-material system.

Assuming the crack propagation area is *dA*, the energy required for crack propagation is provided by the decrease in potential energy of the entire system. Therefore, the energy release rate *G* is defined as:

6 $$G=-\frac{d{E}_{p}}{dA}$$

Assuming the thickness of the bi-material system is δ, then:

7 $$G=-\frac{d{E}_{p}}{\delta d(l-a)}$$

Substituting the total potential energy of the system expressed by Eq. (5), the energy release rate G of the interface crack in the bi-material system can be defined as follows:

8 $$G=-\frac{{F}^{2}}{2\delta }\frac{{A}_{2}{E}_{2}}{{A}_{1}{E}_{1}({A}_{1}{E}_{1}+{A}_{2}{E}_{2})}$$

The above equation can explain the trend of crack derivation, and can also make a comprehensive analysis of the influence of various factors on the energy release rate. Meanwhile, this equation can also be used to evaluate the crack propagation in a single material. For a single material system, *E*_*1*_ = *E*_*2*_ = *E*.

Since the tendency of cracks to develop in the direction of a larger energy release rate, when analyzing TBCs interface deflection cracks, the energy release rate *G* of the crack at the crack initiation point propagating along the interface of the bi-material system and propagating to the ceramic layer can be calculated and compared, then the direction of crack initiation and propagation of the system can be determined.

## Results and discussion

### Analysis of TGO growth behavior

In the process of mixed thermal oxidation, TGO mainly undergoes two morphological changes: thickness thickening and fluctuation increase. TGO creep, uneven distribution of metal elements and different degrees of diffusion increase the fluctuation degree of the interface. The increase in fluctuation degree aggravates the uneven growth of TGO thickness, and this uneven growth will lead to further fluctuation of the interface.

**Figure 5:**
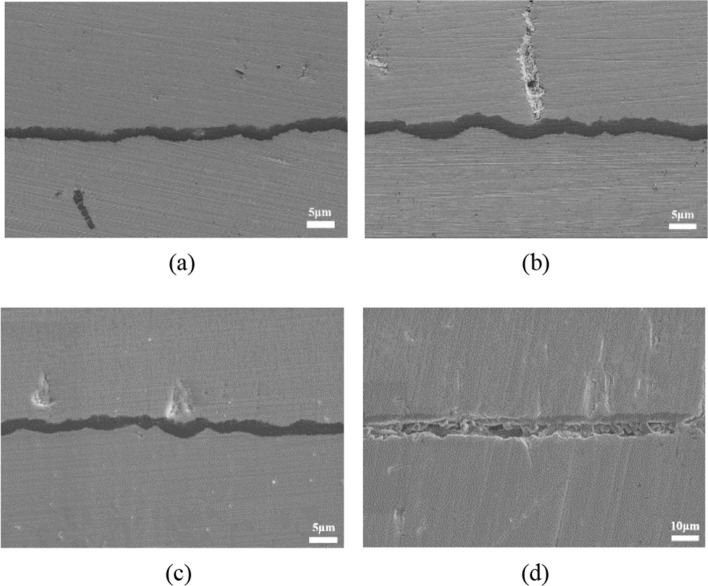
Interface morphology of EB-PVD 8YSZ thermal barrier coating specimen after mixed thermal oxidation at 1100 ℃ (**a**) for 10 h (**b**) for 10 h + 10cycles (**c**) 10 h + 30 cycles (**d**) 10 h + 40 cycles.

Considering the complexity of interface contour changes, this section will provide a specific process for quantifying the thickness and fluctuation degree of TGO and conduct relevant analysis. Figure 5 shows the different interface morphologies of TGO development under mixed thermal oxidation conditions.

It can be seen that the morphology of TGO is mainly composed of two aspects—the degree of fluctuation and the growth thickness. The actual length of TGO in the SEM image is measured by using the software Image J, and its equivalent thickness is calculated. Then, different dimensionless constants are used to define its folding index and two-dimensional roughness, and the corresponding change trend is obtained.

The actual length of TGO is defined as *l*_*d*_, and its specific value is measured in Table 1 below. Then the equivalent thickness* t*_*e*_ can be calculated by the area *A* and the actual length *l*_*d*_, the equation is:

**Table 1:** Actual length *l*_*d*_*,* projection length *l*_*p*_, Normalized Rumpling Index (*NRI*) and equivalent thickness* t*_*e*_ of TGO under different mixed thermal oxidation mode at 1100 °C

Oxidation conditions	l_d_ (μm)	l_p_ (μm)	NRI	t_e_ (μm)
10 h	58.26	57.22	1.015	3.03
10 h + 10 cycles	71.84	69.57	1.033	3.22
10 h + 20 cycles	65.10	60.30	1.080	3.74
10 h + 30 cycles	74.00	63.44	1.166	3.83
10 h + 40 cycles	159.20	126.58	1.258	6.28

9 $${t}_{e}=\frac{A}{{l}_{d}}$$

The fluctuation degree of TGO can be evaluated by the folding index. Ratio of the actual length *l*_*d*_ to the projection length *l*_*p*_ on the x-axis (i.e. image length) can define the normalized rumpling index (*NRI*).

10 $$NRI=\frac{({l}_{d}/{l}_{p}{)}_{aged}}{({l}_{d}/{l}_{p}{)}_{unaged}}$$

For EB-PVD TBCs, in order to improve the deposition efficiency and a obtain better deposition effect, the surface roughness of the substrate must be reduced before deposition, so that the denominator *(l*_*d*_* / l*_*p*_*) *_*unaged*_ value is constant with a constant value of 1. The NRI values of TGO under different thermal oxidation conditions were calculated and are presented in Table 1.

**Figure 6:**
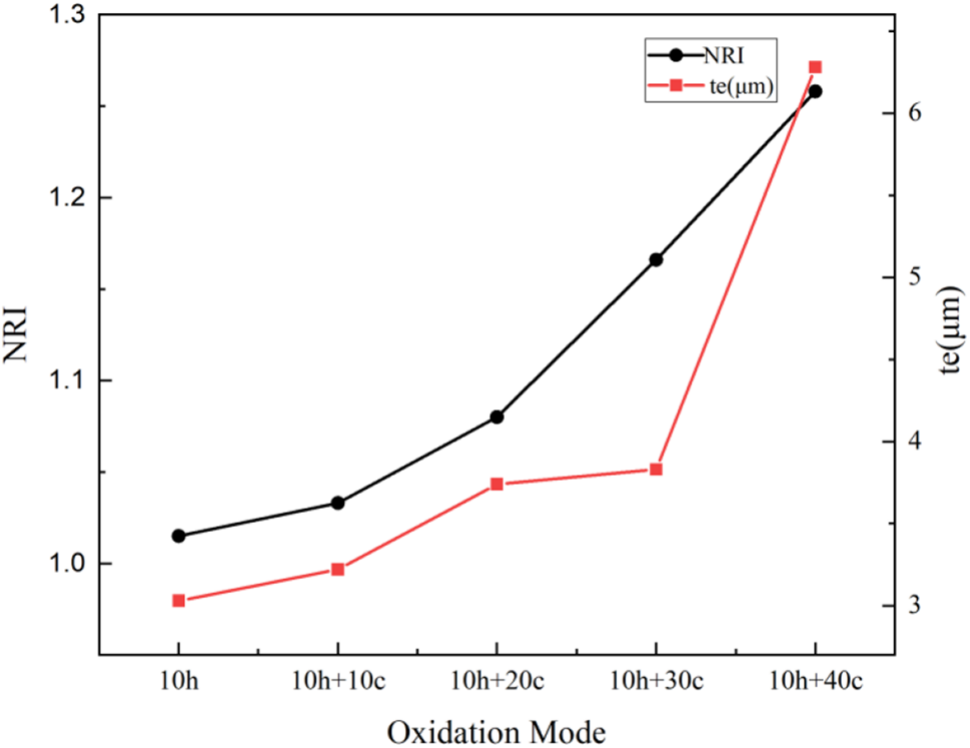
Trend of equivalent thickness *t*_*e*_ and *NRI* of TGO in EB-PVD 8YSZ after different mixed thermal oxidation condition at 1100 °C.

Figure 6 shows the variation trend of *NRI* and equivalent thickness *t*_*e*_. It can be seen from the experimental results that the *NRI* of TGO increases from 1.015 to 1.258, and its equivalent thickness *t*_*e*_ also increases from 3.03 μm to 6.28 μm, showing an upward trend. After 10 h of isothermal oxidation and 20 oxidation cycles, the equivalent thickness of the TGO layer increased only from 3.03 μm to 3.74 μm, while the NRI increased from 1.015 to 1.080. This indicates that TGO is in a state of violent fluctuation, and its main component is still dense Al_2_O_3_, which can release stress by creep.

**Figure 7:**
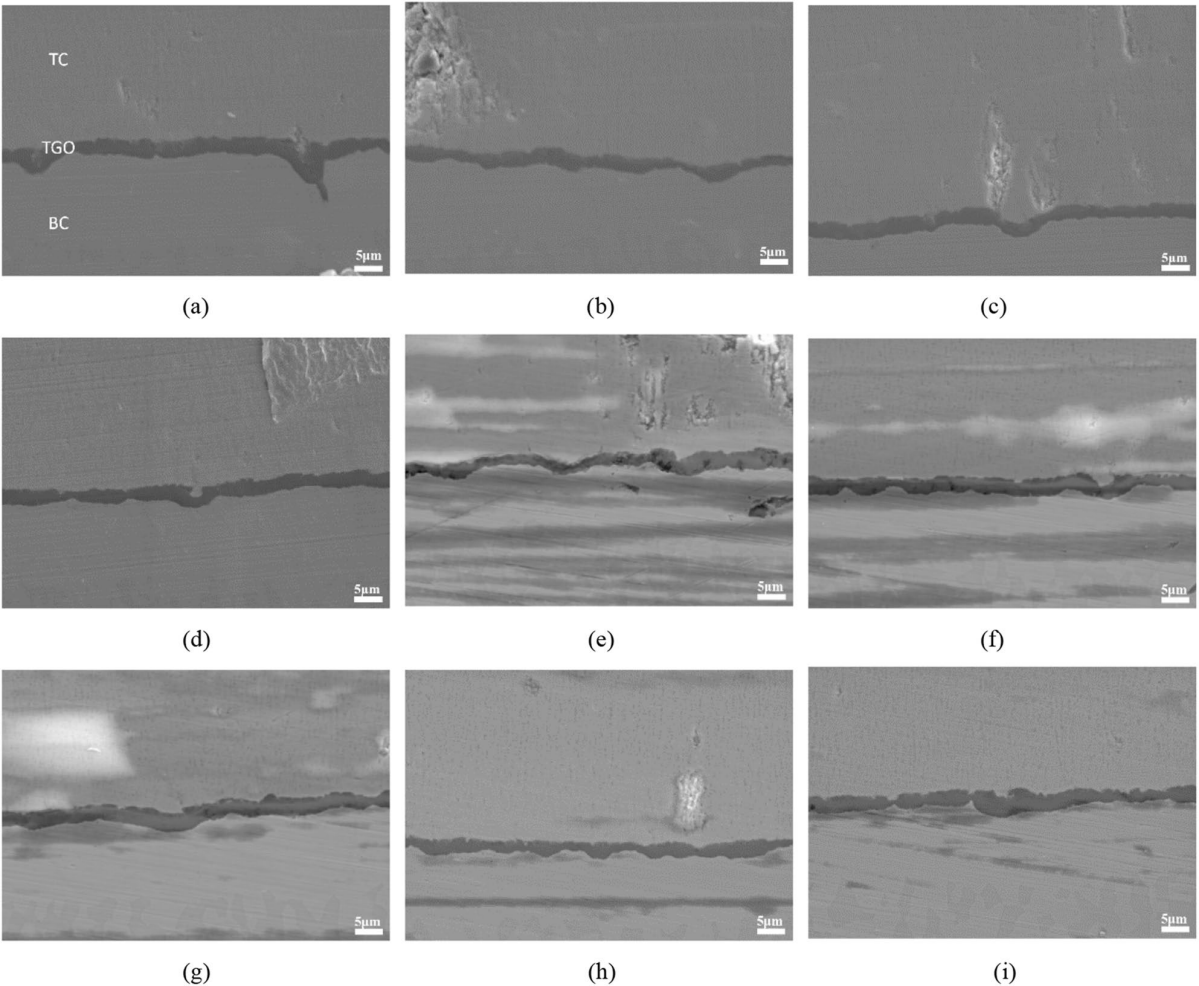
Typical fluctuation and ratcheting of TGO after different heat treatment conditions at 1100 °C (**a**–**d**: 10 h + 25c) (**e**–**g**:10 h + 30c) (**h**–**i**: 10 h + 35c).

The phenomenon of TGO wrinkles and infiltration can be observed from Fig. 7, indicating that ceramic layer will make the ratcheting effect of TGO more obvious, and the mechanical bonding degree between TGO and BC is higher, so the microcracks are more inclined to develop towards the TC. Between (10 h + 30c) and (10 h + 40c), the thickness growth rate of TGO suddenly increases. However, NRI is relatively stable, and there is no noticeable difference between (10 h + 20c) and (10 h + 30c). This indicates that TGO is in the stage of rapid increase in thickness and begins to form loose spinel oxides, the NRI increases mainly due to the formation of spinel oxides. At the same time, the microcracks inside the TBCs increase and the paths that can develop increase, resulting in the occasional shedding of the TGO during the and polishing process.

The two-dimensional roughness index can integrate the fluctuation and thickness of TGO to further measure its degree of change. The average roughness *R*_*a*_, root mean square roughness *RMS* and total roughness height *R*_*t*_ were selected to quantify the morphological changes of TGO. *R*_*a*_ measures the arithmetic mean of the absolute value of the height of the contour in the longitudinal direction from the mean line. RMS calculates the root mean square average of the contour height deviation from the average line (i.e., the standard deviation of the height distribution). They are expressed as:

11 $${R}_{a}=\frac{1}{n}{\sum }_{i=1}^{n}\left|{Z}_{i}\right|$$

12 $$RMS=\sqrt{\frac{1}{n}{\sum }_{i=1}^{n}{Z}_{i}^{2}}$$

where *n* is the number of data points, and *Z*_*i*_ is the distance between the height at point *i* and the average line.

*R*_*t*_ represents the maximum fluctuation range of TGO in the observation area, needs to measure height between the highest peak (*R*_*p*_) and the deepest trough (*R*_*v*_) in the evaluation area.

13 $${R}_{t}=\left|\mathit{max}({R}_{p})\right|+\left|\mathit{max}({R}_{v})\right|$$

The results calculated by Eq. (11)–(13) are shown in Table 2. *R*_*a*_ increased from 1.79 μm to 3.69 μm, *RMS* increased from 1.84 μm to 3.79 μm, and *R*_*t*_ increased from 2.45 μm to 10.33 μm.

**Table 2:** *R*_*a*_, *RMS* and *R*_*t*_ of TGO after different mixed thermal oxidation conditions at 1100 ℃.

Oxidation conditions	Roughness parameters (μm)		
	R_a_	RMS	R_t_
10 h	1.79	1.84	2.45
10 h + 10 cycles	2.46	2.51	5.13
10 h + 20 cycles	2.53	2.69	10.19
10 h + 30 cycles	3.31	3.34	8.36
10 h + 40 cycles	3.69	3.79	10.33

As shown in Table 2, the roughness parameters continue to increase along with the oxidation level. The increase in roughness will cause local stress concentration and reduce the interfacial bonding strength, leading to crack propagation and increasing the possibility of interfacial debonding. However, for the special change of *R*_*t*_, it shows that the peaks and troughs formed by TGO do not fluctuate uniformly, but are inconsistent in depth and have obvious differences in height. It can be inferred that the morphology of TGO is formed by thermal aging. The deeper ' trough ' (inward defect) evolved. All results reflect the increase in interfacial curvature between TC, BC and TGO. The tendency of TBCs to fluctuate and wrinkle is related to the composition and status of TGO. When TGO is mainly composed of Al_2_O_3_, the deformation is dominated by fluctuations. When the composition is mainly composed of loose structures such as spinel oxides, it begins to thicken rapidly.

The evolution of TGO is complex to promote the failure of TBCs, and its change directly or indirectly affects the number of microcracks, the orientation of crack propagation, the size of interfacial fracture toughness and the composition and concentration of residual stress in the coating. Furthermore, the fluctuation and thickening of TGO will also cause considerable residual stress at the interface and promote the formation of cracks. With the growth of TGO, the internal interface of TBCs changes from TC/BC single interface to TC/TGO/BC double interface, which increases the number of defects that generate cracks, and the selectivity of interfacial microcracks is stronger, which will start to derive in the local area with weaker bonding strength. The convergence of microcracks accelerates the propagation of cracks and accelerates the failure of TBCs.

### Analysis of crack propagation behavior

The main reason for the failure of TBCs is the derivation, convergence and interfacial propagation of microcracks. ^25–27^. In this section, the morphologies, especially the interfacial morphology of TBCs after oxidation were observed by SEM. The typical types of interfacial cracks were sorted out and classified, and their derivative characteristics were confirmed. There are mainly the following three typical forms.

#### Longitudinal penetrating cracks

**Figure 8:**
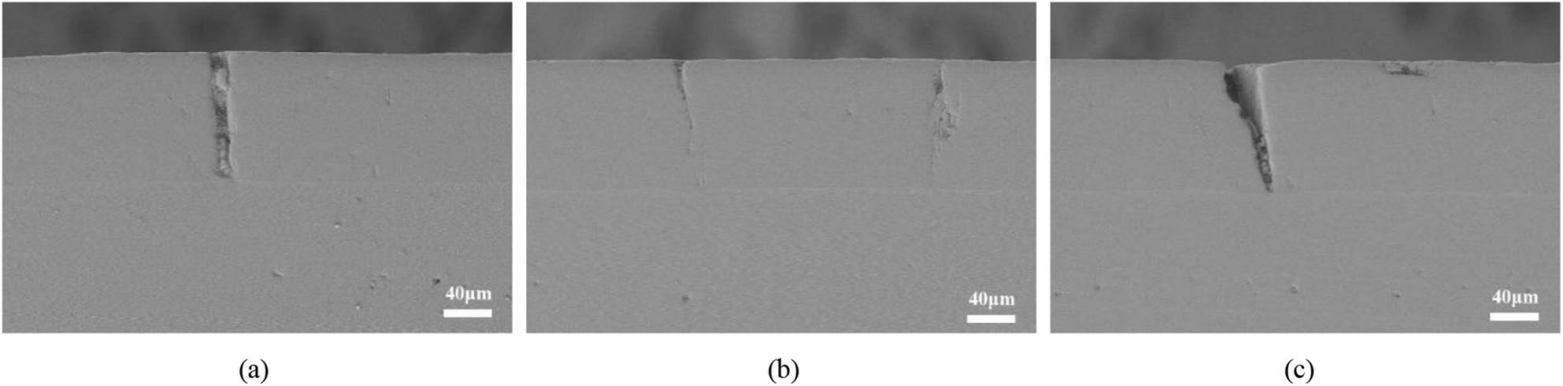
Typical longitudinal crack morphology of EB-PVD TBCs (**a**) developed to the interface (**b**) not develop to the interface (**c**) caused V-shaped cracking of TC.

Figure 8 is a longitudinal crack perpendicular to the interface, which is mainly caused by the repeated traction of external force and the wear of impurities during the preparation of metallographic process, indicating that the bonding strength between columnar crystals of EB-PVD TBCs is significantly lower than its own material strength. Such cracks are present in TC, starting from the surface and may run through the entire thickness direction of the ceramic layer (Fig. 8a), or may stop in the middle of the ceramic layer (Fig. 8b); when the columnar crystals are deformed and squeezed together, a V-shaped crack may occur (Fig. 8c).

#### Lateral propagation cracks

**Figure 9:**
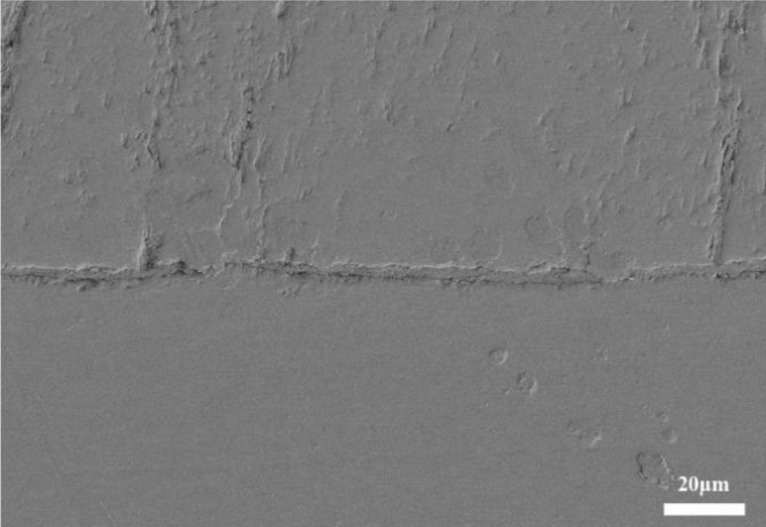
Typical interface lateral propagation crack.

Due to the creep of TGO during oxidation, the mismatch of thermal expansion coefficient between TC/TGO/BC layers and the phenomenon of high-temperature hardening and sintering of TBCs, the interfacial bonding strength is significantly reduced and the toughness is degraded. At the same time, during the process of heating and cooling, the internal accumulated stress further promotes the propagation of microcracks after reaching the critical point at the interface. Figure 9 is a typical interfacial lateral propagation crack, such cracks begin to develop along the area with the lowest bonding strength.

#### Oblique cracks with deflection

**Figure 10:**
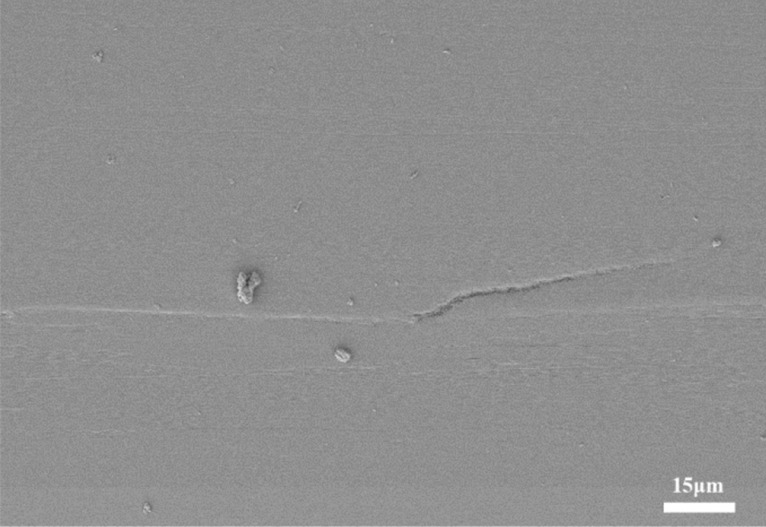
Oblique cracks with deflection.

In fewer cases, the cracks deflected upward into the surface layer and continued to develop along the columnar break-point, as shown in Fig. 10. This kind of oblique crack does not propagate along the lateral part of the interface, that is, the crack initiates at the defects at the BC/TGO interface and develops directly around the TC. In addition, the phenomenon that the oblique cracks cannot develop on the TC surface indicates that the columnar crystal structure of the ceramic layer in EB-PVD TBCs does not run through all the time, but changes with the deposition, so the columnar crystal has the characteristics of segmentation and uneven distribution.

**Figure 11:**
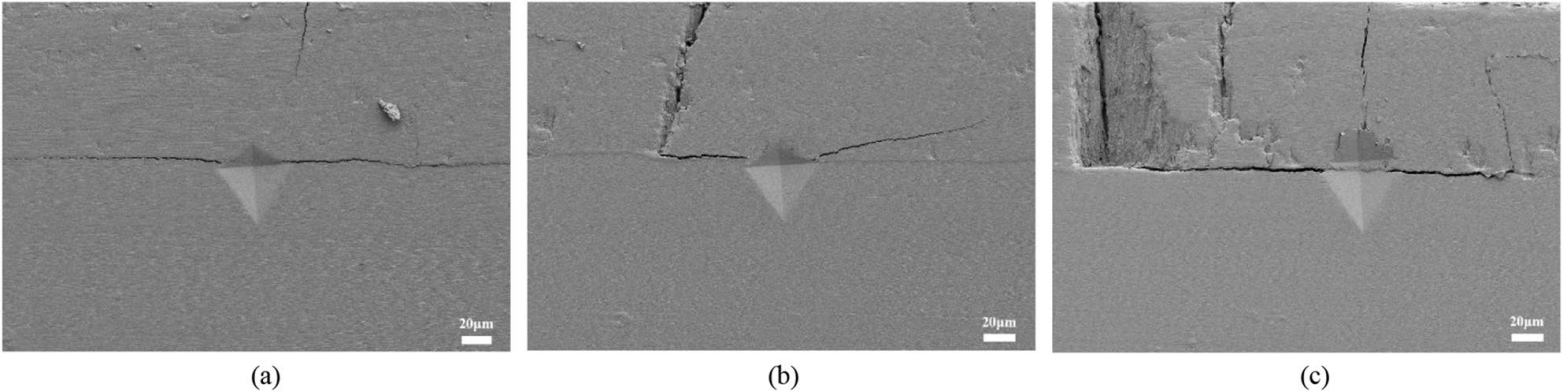
Typical interface crack trend after external force at interface (**a**) Cracking along the interface (**b**) Deflecting into TC (**c**) Causing small-scale cracking.

In order to obtain more interfacial crack samples, sufficient loads are applied at the interface of TBCs. It can be seen from Fig. 11 that without the interference of longitudinal cracks or other defects, the preferred direction of crack development is to develop uniformly along the interface on both sides, followed by deflection to TC, which is the same as the development trend of interface cracks without load. When a lateral crack intersects and competes with a longitudinal crack, it is possible for the lateral crack to stop expanding, or it is also possible to cross the intersection area and continue to move forward. On the contrary, it is not observed that the longitudinal crack penetrates the lateral crack and continues to penetrate the bonding layer. This shows that the longitudinal crack tends to crack along the grain boundary, and the stress intensity required for crack tip propagation is higher than that for lateral. If the lateral crack competes with multiple longitudinal cracks, the columnar crystals in the range of multiple longitudinal cracks may fall off, which is easy to cause a small-scale cracking of the ceramic layer. Further analysis needs to be done by calculating the interface fracture toughness.

Besides, the loading indentation tests also prove the correlation between interfacial roughness and interfacial lateral crack propagation behavior. As the increase of roughness parameters will cause local stress concentration and reduce the interfacial bonding strength, cracks will be easier to propagate at the interface of TBCs. Based on Table 2, the Table 3 below adds information on the length of interfacial cracks under specific load. It can be seen that under the same load of 2.94N at coatings’ interface, the crack length *2a* (note that *2a* means the total length of crack on both sides caused by indentation) are augmented along with the roughness parameters increasing. This further explains that as the oxidation intensifies, the increase in coating roughness parameters leads to stress concentration and bonding strength degradation, and thus makes cracks more prone to propagation.

**Table 3:** Interfacial roughness parameters *vs.* crack lengths with a load of 2.94N at interface.

Oxidation	Roughness parameters (μm)			Crack length (μm)
Conditions	R_a_	RMS	R_t_	2a
10 h	1.79	1.84	2.45	7.38 ± 1.38
10 h + 10 cycles	2.46	2.51	5.13	10.56 ± 2.97
10 h + 20 cycles	2.53	2.69	10.19	18.99 ± 4.52
10 h + 30 cycles	3.31	3.34	8.36	24.32 ± 6.78
10 h + 40 cycles	3.69	3.79	10.33	33.67 ± 10.57

### Analysis of interfacial fracture toughness

It is necessary to explain the selectivity of the interface during lateral crack propagation from the perspective of fracture toughness. Then, the interfacial fracture toughness *K*_*ca*_ of TC/TGO interface and TGO/BC interface in the presence of TGO will be calculated separately, and the fracture toughness *K*_*ca*_ of TC/BC interface in the presence of TGO will be ignored.

Firstly, the average Young’s modulus (*E*) (as shown in Table 4) and hardness (*H*) (as shown in Table 5) of TC, BC and TGO were obtained from multiple point measurements.

**Table 4:** Young’s modulus variation.

E (GPa)	As-received	25 h	50 h	75 h	25 cycles	50 cycles
TC	126.5923	178.2282	156.4559	112.0145	159.5678	178.6793
TGO	–	413.7161	382.8606	224.6272	301.4687	403.4228
BC	230.8842	241.3386	242.5227	127.3924	230.8373	233.6984

**Table 5:** Hardness variation.

H (GPa)	As-received	25 h	50 h	75 h	25 cycles	50 cycles
TC	10.3719	14.1888	9.5543	10.6410	14.2983	9.9428
TGO	–	32.9226	37.5685	20.0772	27.4615	38.9057
BC	7.6247	7.8930	6.4323	5.8502	9.0081	6.3914

**Figure 12:**
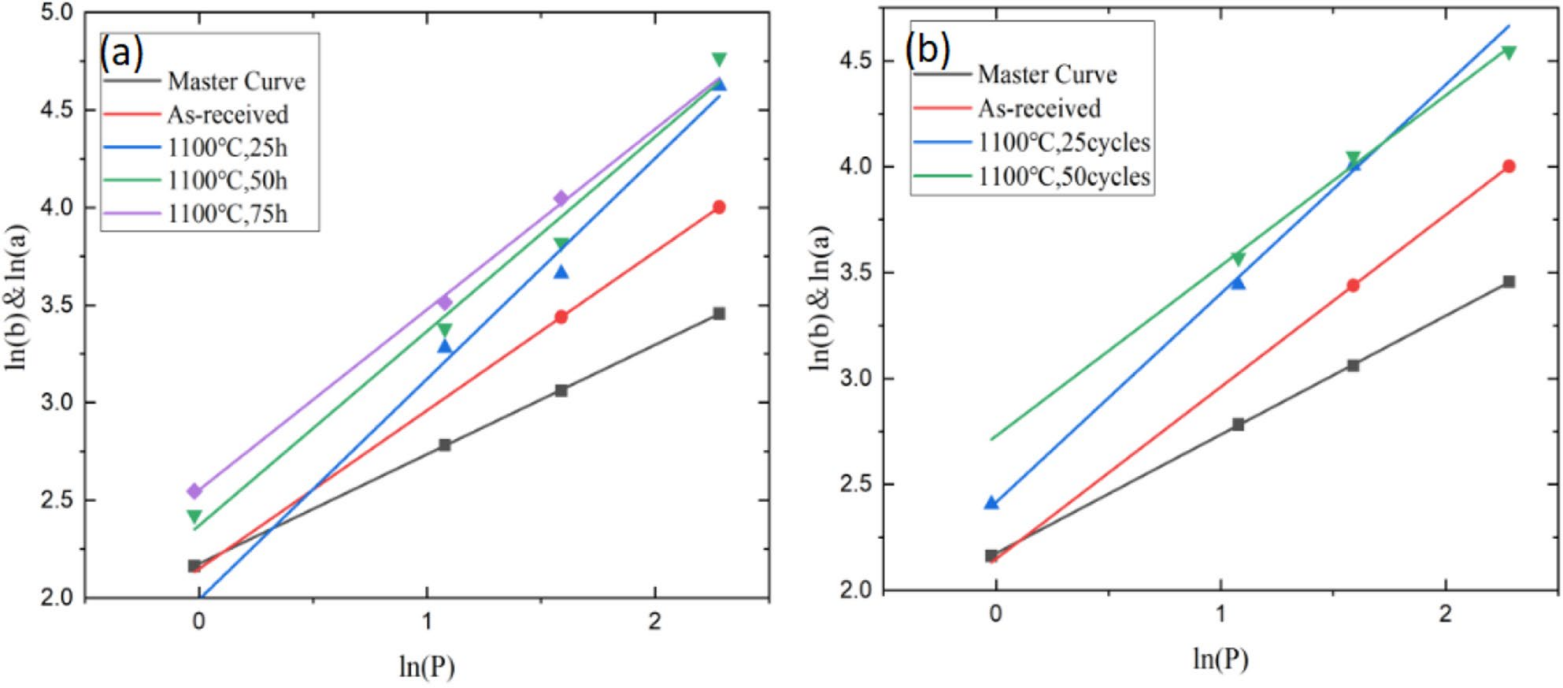
The critical load and critical crack length leading to interface cracking were measured by linear fitting. (**a**) isothermal oxidation (**b**) cyclic oxidation.

Combined with the calculation method of fracture toughness described in Section "Calculation of interfacial fracture toughness", the length of indentation and crack under each load is measured respectively, all the data are linearly fitted by Origin software to obtain the main curve and crack curve. Note that in this evaluation process, 4–5 tests have been made by each load on a certain specimen, but the points shown in Fig. 12 are their average values aiming to plot the logarithmic curves more accurate. The calculation results are summarized in Table 6, it can also be seen that the slopes of the main curve in Fig. 12 are both around 0.5, which is in good agreement with Eq. (2).

**Table 6:** The values of *P*_*c*_, *a*_*c*_ and *K*_*ca*_ under different high-temperature oxidation conditions.

Oxidation Conditions	P_c_/N	a_c_/μm	K_ca_/MPa·m^1/2^
As-received	1.67063	11.73434	2.99927
25 h	0.93807	6.83981	3.68376
25 cycles	0.56802	6.40456	2.26063
50 h	0.35421	4.91711	2.53231
50 cycles	0.10304	2.46024	2.09974
75 h	0.35410	4.91627	1.97878

**Figure 13:**
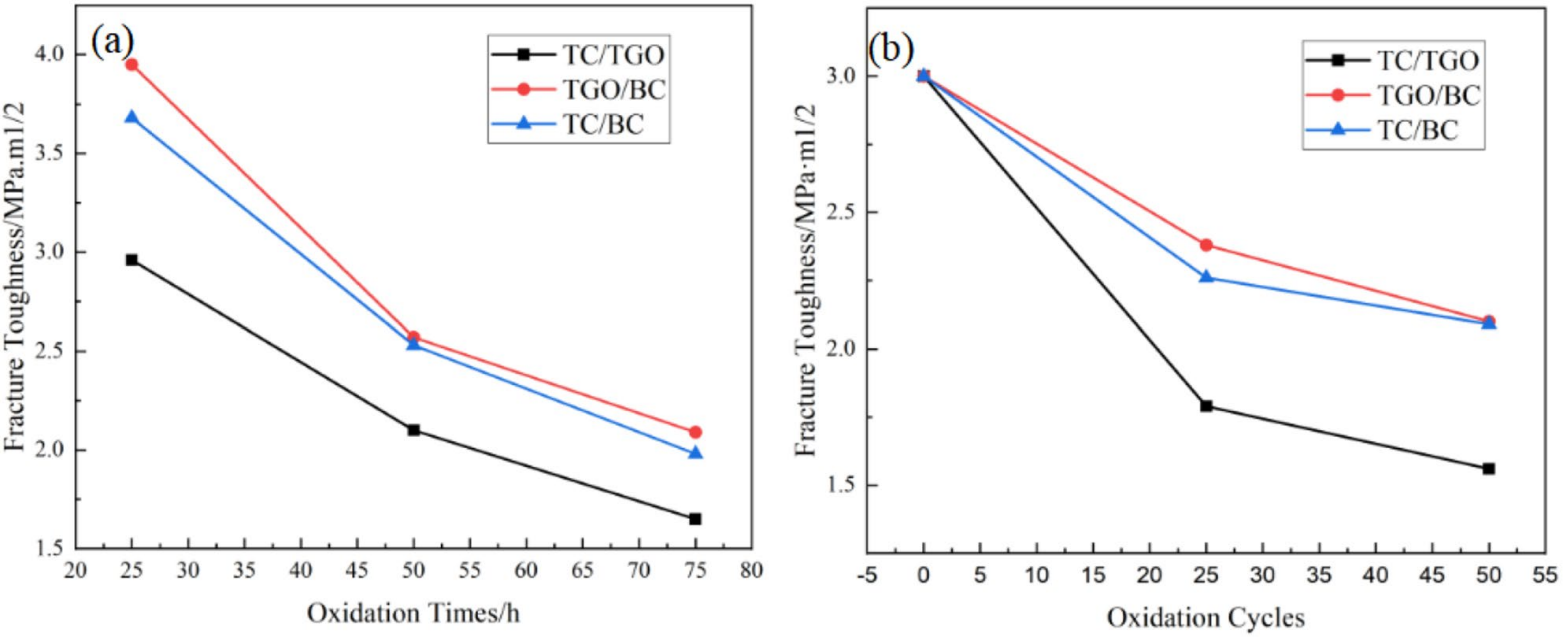
Trend of differernt interfacial fracture toughness of EB-PVD TBCs under 1100 °C (**a**) isothermal oxidation (**b**) cyclic oxidation.

The critical load *P*_*c*_ decreased from 1.67 N to 0.10 N and the critical crack length *a*_*c*_ decreased from 11.73 μm to 2.46 μm. For the specimens after thermal oxidation, *P*_*c*_ decreased with the increase in oxidation temperature, indicating that the ability of the interface to withstand the load before debonding decreases, which means the aging and instability of the interface. In order to further analyze the propagation behavior of TBCs interface cracks, the interfacial fracture toughness of TC/TGO and TGO/BC under different oxidation conditions was tested and calculated, and compared with TC/BC values. The relevant calculation results are summarized in Table 7 and Fig. 13.

**Table 7:** Interfacial fracture toughness of TC/TGO, TGO/BC, TC/BC under different thermal oxidation conditions.

Oxidation Conditions	K_ca_/MPa·m^1/2^		
	TC/BC	TC/TGO	TGO/BC
As-received	2.99927	–	–
25 h	3.68376	2.96291	3.95032
25 cycles	2.26063	1.78850	2.37712
50 h	2.53231	2.10008	2.57289
50 cycles	2.09974	1.56103	2.09553
75 h	1.97878	1.65340	2.08879

With the progress of thermal oxidation, the decreasing trend of the toughness of the three interfaces is consistent, and the decreasing rate of the interfacial fracture toughness gradually decreases, which means that the crack propagation after oxidation becomes easier. In addition, the fracture toughness of TC/TGO is clearly smaller than that of TGO/BC interface, and the fracture toughness of TGO/BC is similar to that of TC/BC ignoring TGO. It can be indicated that the properties of TGO and BC are more similar, the integrity between the two is strong. Coupled with the results of the previous section, there is only a crack type that deflects upward into TC in the interface lateral crack, and there is no type that deflects downward into BC, further proves the interface bonding strength is greater, the possibility of stress mismatch is lower, and the crack tip is more difficult to propagate along the TGO/BC interface. On the contrary, because the fracture toughness of TC/TGO interface is significantly lower than that of TGO/BC, the TC/TGO interface is more prone to stress mismatch, the interface bonding strength is lower, and the crack tends to propagate along this interface.

### Analysis of energy release rate G

For the type of crack that deflects into the ceramic layer (Fig. 10), the cause needs to be analyzed by calculating the energy release rate G. Combined with the theory described in section "Calculation of energy release rate", the calculation method is converted into a TC/BC bi-material system, and the Young 's modulus, cross-sectional area and thickness of TC are defined as *E*_*1*_, *A*_*1*_ and *δ*, respectively. The Young 's modulus and cross-sectional area of BC are *E*_*2*_ and *A*_*2*_, respectively. The thickness of Tc/BC bi-material system is δ′.

When the crack deflects directly into the TC from defect, it can be considered to propagate in a single material. Therefore, the crack propagation energy release rate *G*_*1*_ equation is:

14 $${G}_{1}=-\frac{(F{)}^{2}}{2\delta }\frac{{A}_{2}}{{A}_{1}({A}_{1}{E}_{1}+{A}_{2}{E}_{1})}$$

When the crack expands along the interface and then enters the TC, it is considered that it expands in the TC/BC bi-material system. At this time, the energy release rate *G*_*2*_ of the lateral propagation of the crack along the interface is

15 $${G}_{2}=-\frac{{{F}{\prime}}^{2}}{2{\delta }{\prime}}\frac{{A}_{2}{E}_{2}}{{A}_{1}{E}_{1}({A}_{1}{E}_{1}+{A}_{2}{E}_{2})}$$

For the same crack, the force causing its expansion is equal, i.e. *F* = *F*′. According to the characteristics of crack propagation, cracks inside the material usually propagate along the direction with the largest energy release rate. Therefore, when the crack deflects into the TC, it means that *G*_*1*_ > *G*_*2*_, i.e. *G*_*1*_/*G*_*2*_ > 1. Substituting this into Eq. (14), (15) we get:

16 $$\frac{{\delta }{\prime}}{\delta }\frac{{E}_{1}}{{E}_{2}}\frac{{A}_{1}{E}_{1}+{A}_{2}{E}_{2}}{{A}_{1}{E}_{1}+{A}_{2}{E}_{1}}>1$$

The parameters *A*_*1*_*, A*_*2*_, thickness *δ*, *δ'* of TBCs in this paper are all known quantities, and the conversion of Eq. (16) can be obtained:

17 $${E}_{1}>0.5{E}_{2}$$

Equation (17) can be used to estimate whether the cracks in EB-PVD TBCs have the possibility to propagate to TC at a certain time, which can well explain why the cracks generated inside TBCs do not propagate along the TC/TGO interface with lower bonding strength. It can also explain why there is no definite oxidation time or cycle number for the generation of inclined cracks propagating to TC. In the process of high-temperature oxidation, the internal microstructure and oxidation characteristics of TBCs determine that its uniformity is relatively poor, which is embodied in the anisotropic columnar crystal structure in TC and the change of TGO composition during oxidation. Therefore, the measured Young's modulus and hardness have discrete characteristics.

It is worth noting that even under the same oxidation time, the surrounding micro-environment and performance characteristics of the defects that cause cracks are not the same. Taking the TBCs specimens after isothermal oxidation at 1100 °C for 25 h as an example, the average Young 's modulus *E*_*1*_ of TC is 178.23 GPa, and the average Young’s modulus *E*_*2*_ of BC is 370.25 GPa, E_1_ < 0.5E_2_. However, at other measurement points, the Young’s modulus of BC is only 241.34 GPa, and E_1_ > 0.5E_2_. If there is a defect that can cause a crack at this measurement point, the crack may not choose to propagate along the interface, but instead deflect directly into the TC.

Consequently, for EB-PVD TBCs, only when the Young’s modulus of TC and BC near the crack origin satisfies the specific relationship, cracks can extend into TC. In addition, cracks tend to develop along the cluster breakpoints due to the hindrance of columnar crystals, so such cracks usually cannot develop to the surface of TC or cause large-scale cracking. It can be proved that the lateral cracks developed along TC/TGO are still the main cause of TBCs failure, in order to improve the stability of TBCs from the perspective of resisting crack propagation, it is still necessary to improve the interface fracture toughness.

## Conclusion

In this paper, EB-PVD 8YSZ TBCs were investigated under different oxidation conditions at 1100 °C. The interfacial morphology of the specimens after oxidation was analyzed and the interfacial crack propagation methods were classified. The evolution of TGO was summarized and quantified. Based on the evolution of TGO, the reasons for the formation of the interfacial propagation behavior were analyzed. The main conclusions are as follows:

1. The typical interfacial cracks formed by EB-PVD TBCs after oxidation mainly include three forms: vertical cracks present only in TC (including small scale V-shaped spalling); lateral cracks propagating along the interface initiated by interface defects; and oblique cracks deflecting to the TC.
2. The morphological evolution of TGO is complex, and the change of TGO is characterized by equivalent thickness (*t*_*e*_), normalized rumpling index (*NRI*) and two-dimensional roughness index (*R*_*t*_,* R*_*a*_, *RMS*). The results show that TGO increases the quantity, complexity and inhomogeneity of interfaces in TBCs, changes the internal structure of TBCs and affects the number and propagation direction of interfacial cracks.
3. Under the influence of TGO evolution, the development trend of interfacial lateral cracks follows certain rules, which are explained by the interfacial fracture toughness *K*_*ca*_ and energy release rate *G*. The results show that TGO reduces the fracture toughness of TC/BC, changes the energy release rate required for crack propagation at the interface, and makes the interface crack derivative trend show the characteristics of lateral expansion along TC/TGO and deflection to the TC. Since the cracks induced by the energy release rate to TC is limited by the columnar crystal, the lateral cracks propagation along the TC/TGO interface is the main reason for the failure of TBCs.

## Data Availability

The data that support the findings of this study are available from the corresponding author, upon reasonable request.
